# *Escherichia coli*–expressed near full length HIV-1 envelope glycoprotein is a highly sensitive and specific diagnostic antigen

**DOI:** 10.1186/1471-2334-12-325

**Published:** 2012-11-27

**Authors:** Sheikh M Talha, Satish Kumar Nemani, Teppo Salminen, Sushil Kumar, Sathyamangalam Swaminathan, Tero Soukka, Kim Pettersson, Navin Khanna

**Affiliations:** 1 Department of Biotechnology, University of Turku, 20520, Turku, Finland; 2 Recombinant Gene Products Group, International Centre for Genetic Engineering & Biotechnology, Aruna Asaf Ali Marg, New Delhi, 110067, India

**Keywords:** Recombinant HIV-1 envelope glycoprotein, Expression in *E. coli*, Eu^3+^-chelate, Time-resolved fluorescence, Time resolved fluorometry, HIV diagnostics

## Abstract

**Background:**

The Human Immunodeficiency Virus type 1 (HIV-1) envelope glycoprotein gp160, useful in detecting anti-HIV-1 antibodies, is difficult to express in heterologous hosts. The major hurdles are its signal sequence, strong hydrophobic regions and heavy glycosylation. While it has not been possible to express full length recombinant (r)-gp160 in *E. coli*, it can be expressed in insect and mammalian cells, but at relatively higher cost. In this work, we report *E. coli*-based over-expression of r-gp160 variant and evaluate its performance in diagnostic immunoassays for the detection of anti-HIV-1 antibodies.

**Methods:**

A deletion variant of r-gp160 lacking hydrophobic regions of the parent full length molecule was expressed in *E. coli* and purified to near homogeneity using single-step Ni(II)-affinity chromatography. Biotinylated and europium(III) chelate-labeled versions of this antigen were used to set up one- and two-step time-resolved fluorometric double antigen sandwich assays. The performance of these assays was evaluated against a collection of well-characterized human sera (n=131), that included an in-house panel and four commercially procured panels.

**Results:**

In-frame deletion of three hydrophobic regions, spanning amino acid residues 1–43, 519–538 and 676–706, of full length HIV-1 gp160 resulted in its expression in *E. coli*. Both the one- and two-step assays manifested high sensitivity unambiguously identifying 75/77 and 77/77 HIV-1 positive sera, respectively. Both assays also identified all 52 HIV-seronegative sera correctly. Between the two assays, the mean signal-to-cutoff value of the two-step assay was an order of magnitude greater than that of the one-step assay. Both assays were highly specific manifesting no cross-reactivity towards antibodies specific to other viruses like hepatitis B, C, and human T cell leukemia viruses.

**Conclusions:**

This study has demonstrated the expression of r-gp160 variant in *E. coli,* by deletion of hydrophobic regions, and its purification in reasonable yields. This underscores the potential for cost saving in antigen production. Evaluation of this antigen in a double antigen sandwich two-step assay showed it to be a highly sensitive and specific HIV-1 diagnostic reagent. The amenability of this assay to the one-step format suggests its potential utility in developing a rapid point-of-care HIV-1 diagnostic test.

## Background

Human immunodeficiency virus (HIV), a member of the family *Retroviridae*, is the causative agent of acquired immunodeficiency syndrome (AIDS), characterized by profound immune system dysfunction [1]. Recent estimates of the global AIDS burden indicate that there were 2.7 million new infections (bringing the total number to ~34 million infected individuals worldwide) and 1.8 million deaths in 2010 [2]. Diagnostic tests play a critical role in curtailing the spread of HIV, through early detection of infection to identify individuals needing antiviral therapy, and in screening blood and blood related products [3-5]. Most commonly used HIV screening tests are based on the principle of enzyme immunoassay (EIA) that detect either the antibodies (first, second and third generation EIAs) or p24 antigen as well (fourth generation EIA) [6,7]. The envelope glycoprotein (gp160) of HIV-1 is well documented as a viral antigen that presents immunodominant regions for serodiagnosis of HIV-1 infections [8-11]. Native gp160 which can be obtained by HIV-1 infection of H9 cells in tissue culture [12] has been shown to detect earliest HIV-1 seroconversion in different EIAs [12,13]. The inherent biohazard entailed in gp160 production can be circumvented through recombinant expression. However, the gp160 antigen, with its strong hydrophobic regions and heavy glycosylation, is a difficult candidate for heterologous expression and purification [8,14,15]. Nevertheless, recombinant (r)-gp160 can be expressed in insect and mammalian cells, and purified by immunoaffinity or lectin affinity chromatography [14-18]. Though both native and r-gp160 are commercially available, the expensive expression and purification systems increase the production cost of the protein, and in turn contribute to increased cost of HIV testing.

One way to cut down the cost of HIV testing would be to try and exploit the high production potential of prokaryotic expression hosts to produce r-gp160. However, full-length r-gp160 expression in *E. coli* has not been successful. It was reported that a plasmid containing full-length HIV-1 gp160 insert was toxic to *E. coli* resulting in either elimination or rearrangement of the insert [19]. It has been reported that there are two hydrophobic regions in the C-terminal half of gp160 [8]. In this study, we deleted these two regions, as well as the hydrophobic signal peptide, implicated in low expression in mammalian cells [14,15], to create a deletion mutant, Δgp160, and found this to be expressed quite well in *E. coli*, leading to the purification of 15–20 mg r-Δgp160 antigen per liter of induced culture. Compared to the native full-length gp160, the r-Δgp160 antigen developed in this study is almost full-length, lacking just ~10% of the parent sequences (representing three hydrophobic regions). We used two versions of this antigen to set up one- and two-step time-resolved fluorometric (TRF) double antigen sandwich (bridge) assays as described earlier [20]. One version of the antigen was biotinylated [immobilized on streptavidin (SA)-coated microtiter wells, to capture anti-HIV antibodies] and the other, labeled with a europium (Eu^3+^) chelate (as a tracer to reveal bound antibodies). By testing a collection of well-characterized human sera (n=131) in these assays, we provide evidence to show that the *E.coli*-expressed r-Δgp160 antigen is a potential diagnostic tool of high sensitivity and specificity for the detection of anti-HIV-1 antibodies.

## Methods

### Materials

*E. coli* host strains DH5α and BL21(DE3), used for cloning and expression, respectively, were purchased from Invitrogen Life Technologies (Carlsbad, CA, USA). Plasmid pET-28a(+) was from Novagen (Madison, WI, USA). Plasmids pTrxBAP and pBirA have been described before [20,21]. Enzymes for routine cloning work were procured from MBI Fermentas (Burlington, Canada). Ni-NTA super flow resin and isopropyl-β-D-thiogalactopyranoside (IPTG) were from Qiagen (Hilden, Germany), and Calbiochem-EMD Biosciences (La Jolla, CA, USA), respectively. The intrinsically fluorescent isothiocyanate activated nonadentate Eu^3+^-chelate [22,23] and biotin isothiocyanate [24] for antigen labeling were synthesized as before. Nucleic Acid Purification (NAP) Sephadex G-25 columns were procured from GE Healthcare (Uppsala, Sweden). SA-coated normal capacity low-fluorescent microtiter plates were obtained from Kaivogen Oy (Turku, Finland).

A collection of 131 human sera samples was assembled for the study. This consisted of one in-house panel and 4 commercially procured panels. The in-house panel of 59 sera samples (consisting of 22 HIV positive and 37 HIV negative sera), pre-screened using Vidas HIV Duo Quick kit (bioMérieux SA, Marcy I’Etoile, France), was obtained from the Department of Virology, University of Turku. These sera samples were collected as per the guidelines of Ethics Committee of the Turku University Hospital. Well-characterized worldwide HIV performance panel (WWRB 302–01 to WWRB 302–30), HIV-1 seroconversion panel (PRB 931–01 to PRB 931–09), anti-HIV-1 low titer performance panel (PRB 108–1 to PRB 108–15) and viral co-infection panel (PCA 201–01 to PCA 201–25) were purchased from SeraCare Life Sciences (Milford, MA, USA). In total, there were 77 anti-HIV-1 positive, 2 anti-HIV-2 positive and 52 anti-HIV negative samples.

### Generation of recombinant gp160 antigens

The 760 amino acid (aa) residue long r-Δgp160 antigen was designed by eliminating the signal sequence (aa 1–43) and two internal hydrophobic regions (aa 519–538 and 676–706) of HIV-1 (strain NL4-3). The corresponding synthetic gene, codon-optimized for *E. coli* expression was obtained from Geneart (Regensburg, Germany), and used to express two variants of the encoded recombinant antigen. This synthetic gene was inserted into (i) pET-28a(+), in-frame with the vector-encoded 6x-His tag and stop codon at its carboxyl end, to express r-Δgp160 antigen, and (ii) pTrxBAP in frame with the vector-encoded Trx (thioredoxin)-6x-His tag-BAP sequences at the amino terminus, to express the r-Trx-BAP-Δgp160 antigen. The resultant plasmids were introduced separately into *E. coli* BL21(DE3), and induced to express with 1 mM IPTG. In order to achieve biotinylation of the r-Trx-BAP-Δgp160 antigen *in vivo*, *E. coli* cells expressing this antigen were co-transformed with IPTG-inducible biotin ligase expressing plasmid pBirA and cultured in medium supplemented with biotin (10 μg/ml).

As both recombinant protein variants were associated with the insoluble fraction, they were purified from induced cells under denaturing conditions [20]. Briefly, induced cell pellets were re-suspended in lysis buffer (6 M guanidine HCl/20 mM Tris–HCl/300 mM NaCl/10% glycerol/0.1% sodium deoxycholate/1% Tween-20/10 mM β-mercaptoethanol/20 mM imidazole, pH 8) and lysed by sonication at 4°C (Sonics Vibracell sonicator). The resulting lysate was clarified and chromatographed on a 5 ml Ni-NTA super flow resin column. After washing the column (wash buffer was similar to lysis buffer lacking sodium deoxycholate with the exception that 6 M Guanidine-HCl was replaced by 8 M urea), elution was performed using a linear imidazole gradient (20–500 mM) in wash buffer. All the eluted fractions were analyzed by SDS–PAGE, the peak fractions were pooled together, filtered through 0.22 μm membrane and stored in 1 ml aliquots at a concentration of about 1 mg/ml at −20°C until further use. One aliquot was thawed at a time, heat-treated at 80°C for 90 seconds, snap-cooled in ice bath and the buffer was changed to 50 mM sodium carbonate buffer, pH 9.8 using NAP-5 or NAP-10 column, for subsequent labeling reaction.

### Preparation of labeled antigens

The r-Δgp160 antigen was labeled with a 30-fold molar excess of Eu^3+^-chelate at 4°C for 16–20 hours in 50 mM sodium carbonate buffer, pH 9.8, essentially as described before [20]. The r-Trx-BAP-Δgp160 (which failed to be efficiently biotinylated *in vivo*) was *in vitro* (chemically) biotinylated with a 40-fold molar excess of biotin isothiocyanate. The reaction was performed in 50 mM sodium carbonate buffer, pH 9.8, at room temperature (RT) for 4 hours. Both the unincorporated labels were removed by passing the reaction mixtures sequentially through NAP-5 and NAP-10 columns. For the sake of simplicity, Eu^3+^-chelate labeled r-Δgp160 and chemically biotinylated r-Trx-BAP-Δgp160 antigens are referred to as r-Δgp160-Eu^3+^ and r-Δgp160-Bio, respectively. The labeled antigens were supplemented with 0.1% bovine serum albumin and 0.05% sodium azide, filtered through 0.22 μm membranes and stored at 4°C, until further use.

### In-house TRF double antigen sandwich assays

Either r-Δgp160-Bio antigen (600 ng/100 μl assay buffer/well) or the previously reported [20]*in vivo* biotinylated r-Bio-HIV-1env antigen (200 ng/100 μl assay buffer/well) was immobilized on SA-coated microtiter wells. The assay buffer was 50 mM sodium carbonate, pH 9.6 (containing 25 mM NaCl/0.1% Tween-20/2.5% BSA/1.25% sucrose/0.06% γ-globulins from bovine blood/0.05% NaN_3_). Binding of biotinylated antigen to SA-coated microtiter wells was allowed to proceed for 1 hour at RT with shaking. The wells were then rinsed twice with wash buffer (154 mM NaCl/0.5 M KCl/0.1% Tween-20/50 mM potassium phosphate buffer, pH 7.2). These antigen-coated wells were used in one- and two-step assay formats as described below.

In the one-step assay (performed only with r-Δgp160-Bio antigen-coated well), a mixture of 50 μl serum, diluted (1:10) in assay buffer, plus 50 μl assay buffer containing 100 ng r-Δgp160-Eu^3+^, was added to each well and incubated for 1 hour at RT with shaking. The wells were washed 7 times with wash buffer. Time-resolved fluorescence for Eu^3+^-chelate was measured (λ_ex_:340nm; λ_em_:615nm) from dry wells using Victor^3^V 1420 Multilabel counter (modified standard europium protocol; measurement height 5 mm).

In the two-step assay, 100 μl serum (diluted 1:20 in assay buffer) was added into each well and incubated for 1 hour at RT with shaking. Following 4 washes, 100 μl assay buffer containing either 100 ng r-Δgp160-Eu^3+^ (where r-Δgp160-Bio was immobilized as capture antigen) or r-HIV-1env-Eu^3+^ (where r-Bio-HIV-1env was immobilized as capture antigen) was added to each well and incubated for 1 hour at RT with shaking. The wells were washed 7 times with wash buffer and read as above.

### Data analysis

Each assay was evaluated with all the sera on the same day and the results obtained from different assays in single analysis were compared. Sera were designated as either positive or negative using cutoff values of 1000 counts per second (cps) and 924 cps, respectively for the one- and two-step r-Δgp160-based assays developed in this study. A cutoff value of 818 cps was used for the two-step r-HIV-1env assay, based on the previous study [20]. These cutoff values were obtained by adding 3 times the standard deviation (SD) to the mean read-out of 37 HIV-negative sera (of the in-house panel) in the one-step r-Δgp160- (mean = 598 cps; SD = 134 cps), two-step r-Δgp160- (mean = 509 cps; SD = 138 cps), and two-step r-HIV-1env- (mean = 577 cps; SD = 80 cps) antigen based assays described above. The use of three times SD to determine the cutoff value was based on a preliminary characterization of the assay with seronegative samples (n=37). Serum samples with ‘signal-to-cutoff’ (S/Co) ratios of <1.0 were designated as negative, while those with S/Co≥1.0 were designated as positive. Statistical significance of differences in sensitivities between assays was assessed using two-tailed Student’s t test. Differences were considered to be of statistical significance when the probability (p) was <0.05.

## Results and discussion

### Recombinant gp160 antigens

**Figure 1:**
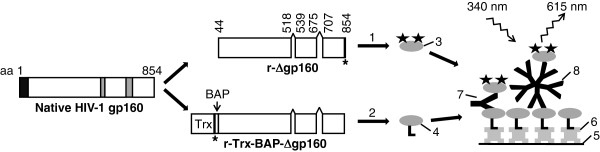
**Design of r-Δgp160 and r-Trx-BAP-Δgp160 antigens and TRF assay**. The left part of the figure shows a schematic representation of the native HIV-1 gp160 molecule. The black and grey boxes denote the signal sequence and the two internal hydrophobic regions in the C-terminal half of native gp160, respectively. Depicted in the middle part of the figure are the two recombinant antigens created for this study, r-Δgp160 and r-Trx-BAP-Δgp160. Both lack the signal sequence (black box) and the two internal hydrophobic regions (grey boxes) of native gp160. An additional sequence consisting of thioredoxin (Trx) and biotin acceptor peptide (BAP) was fused to the N-terminus in r-Trx-BAP-Δgp160. Both proteins were provided with 6x-His tags whose positions are indicated by the asterisks. The numbers indicate the starting and ending aa residues of the native gp160 sequences retained, and fused in-frame, in the recombinant antigens. Shown to the right is design of the double antigen sandwich assay: the r-Δgp160 and r-Trx-BAP-Δgp160 antigens were labeled with Eu^3+^-chelate and biotin isothiocyanate, respectively. These labeling reactions (denoted by 1 and 2) produced r-Δgp160-Eu^3+^ (3) and r-Δgp160-Bio (4). The biotinylated capture antigen r-Δgp160-Bio was bound to SA (6) coating the microtiter well surface (5). Serum anti-HIV-1 IgG (7) and IgM (8) that are captured in the wells are revealed using the tracer, r-Δgp160-Eu^3+^ (3), and visualized by excitation at 340 nm followed by time-resolved measurement of fluorescence at 615 nm.

**Figure 2:**
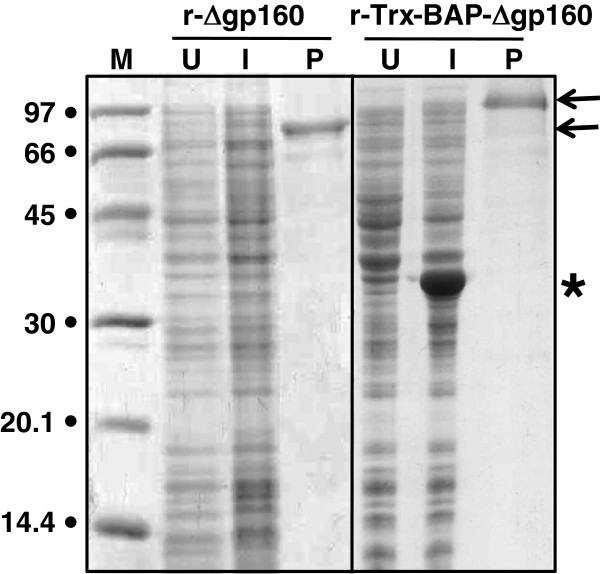
**SDS-PAGE analysis of r-Δgp160 and r-Trx-BAP-Δgp160 antigens.** Aliquots of total lysates of *E. coli* harboring the r-Δgp160 and r-Trx-BAP-Δgp160 antigen constructs, before (lanes ‘U’) and after (lanes ‘I’) IPTG induction, and aliquots of the affinity-purified antigens (lanes ‘P’), were electrophoresed on denaturing gels and visualized by Coomassie staining. Protein size markers were run in lane ‘M’; their sizes (in kDa) are shown on the left. The arrows shown on the right denote r-Trx-BAP-Δgp160 (upper) and r-Δgp160 (lower), respectively. The asterisk denotes the position of biotin ligase enzyme.

The two HIV-1 gp160 antigen variants, r-Δgp160 and r-Trx-BAP-Δgp160, created for this study are shown schematically in Figure 1. Both were designed to delete the amino-terminal signal peptide and two internal hydrophobic stretches of the native HIV-1 gp160 molecule without disruption of the reading frame. Additional sequences were added to these through the use of distinct expression vectors. Both were provided with 6x-His tags to facilitate purification. The key difference between the two was that the r-Trx-BAP-Δgp160 variant also contained at its amino terminus a thioredoxin (Trx)-biotin acceptor peptide (BAP) fusion partner. The purpose of the Trx moiety was to promote solubility of the recombinant antigen and thus facilitate its *in vivo* biotinylation at the target site provided by BAP. The two antigens were expressed by IPTG induction of BL21(DE3) host cells harboring the cognate expression constructs. In the case of r-Trx-BAP-Δgp160, bacterial biotin ligase was co-expressed to mediate biotinylation of its BAP moiety. An SDS-PAGE analysis of aliquots of cells before and after induction of expression of the r-Δgp160 and r-Trx-BAP-Δgp160 constructs is shown in Figure 2. It was difficult to discern the presence of a distinct induced polypeptide of the predicted molecular weights in both cases, though an additional band of ~34 kDa, consistent with the size of biotin ligase, was clearly seen in cells harboring the r-Trx-BAP-Δgp160 construct. Immunoblot analyses of these induced lysates with a monoclonal antibody specific to the 6x-His tag revealed that both r-Δgp160 and r-Trx-BAP-Δgp160 antigens are indeed present in the induced lysates. However, both recombinant antigens were found to be associated with the insoluble fraction (data not shown). Apparently, the Trx fusion is ineffective in promoting solubility of r-Δgp160 antigen. This is consistent with the previous observations that *E. coli*-expressed Trx fusion antigens are not necessarily soluble [20,21]. Therefore, we attempted to purify these antigens under denaturing conditions. This resulted in near homogenous preparations of the two antigens, as evidenced by the SDS-PAGE profiles of the pooled peak fractions in Figure 2 (lanes marked ‘P’). The observed mobilities of purified r-Δgp160 and r-Trx-BAP-Δgp160 were consistent with their predicted sizes of ~88 and ~109 kDa, respectively. Our data show that deletion of the hydrophobic regions is compatible with the expression of r-Δgp160 variants in *E. coli*. Starting from 1L induced *E. coli* cultures we obtained ~20 and ~15 mg, respectively, of purified r-Δgp160 and r-Trx-BAP-Δgp160. However, as purifications were carried out in the presence of urea, it had to be removed from the purified antigens prior to their labeling for use in the double antigen sandwich assays (below). We did not determine if the purified proteins are correctly folded and retained the integrity of their conformational epitopes. However, the recognition of linear epitopes by their cognate antibodies may be sufficient to develop a sensitive immunoassay. Further, the hydrophobic regions deleted in r-Δgp160 are not exposed in the native structure of the full-length protein and therefore not expected to contribute to immunoreactivity [25]. It is relevant to point out that expression of full-length HIV-1 gp160 in *E. coli* has not been possible thus far due to instability/toxicity issues related to full-length, in-frame gp160 gene [19]. In mammalian cells, gp160 undergoes proteolysis in the Golgi complex to produce gp120 and gp41. It was shown recently that when gp120 is expressed in the absence of gp41 in mammalian cells it forms aberrant disulfide linked dimers [26]. Further, it has been reported that gp41 which can be expressed in *E. coli*, is associated with inclusion bodies regardless of whether it has a GST fusion partner [27,28]. Purification under denaturing conditions was reported to yield ~5 mg [28] to ~12 mg [27] soluble protein/L of bacterial cell culture.

### Design of double antigen sandwich TRF assays

Having obtained the pair of gp160-based recombinant antigens described above, we set up a TRF double antigen sandwich assay [20]. In this instance, r-Trx-BAP-Δgp160 was immobilized onto SA-coated plates and used to capture anti-HIV antibodies, and r-Δgp160-Eu^3+^ was employed in TRF assay to detect the captured antibodies. We have found in earlier work that immobilizing the biotinylated capture antigen on SA-coated plate enhances the sensitivity of the assay [29]. Surprisingly, initial assays revealed that this assay was untenable as it required very high amounts of r-Trx-BAP-Δgp160 antigen to obtain acceptable signal to background ratios. Further investigation showed this to be a result of low levels of *in vivo* biotinylation of this antigen (data not shown). The reason for this is not clear, though presumably it may be a reflection of the large size of the antigen, about twice as large as the *in vivo* biotinylated HIV-1 and −2 gp160 deletion variants reported earlier [20], and its intrinsic insolubility. It may also have been due to inaccessibility of the BAP moiety placed between the Trx and Δgp160 components of the chimeric protein. However, we think this is unlikely as similar internal positioning of BAP did not compromise biotinylation in our earlier studies [20,21]. To compensate for the lack of *in vivo* biotinylation, we resorted to *in vitro* biotinylation of this antigen. The resultant *in vitro* biotinylated derivative, r-Δgp160-Bio, was used (in place of the initially intended r-Trx-BAP-Δgp160) in the double antigen sandwich TRF assay outlined above (Figure 1). Using r-Δgp160-Bio antigen immobilized on SA-coated plates, we tested sera for anti-HIV antibodies using either a one-step or two-step assay protocol. In the former, serum and r-Δgp160-Eu^3+^ antigen were added together into the microtiter well containing SA-immobilized r-Δgp160-Bio, whereas in the latter a wash step was introduced after serum addition, and then followed by the addition of the r-Δgp160-Eu^3+^ antigen. The performance of both these assays utilizing the r-Δgp160 antigen pair above, against a large panel of well-characterized sera (n=131) was analyzed as described below.

### Performance evaluation of the r-Δgp160-based one- and two-step double antigen sandwich TRF assays

**Figure 3:**
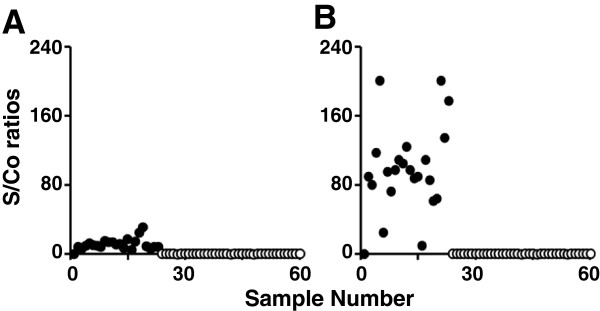
**Analysis of an in-house sera panel using the one- and two-step double antigen sandwich TRF assays.** A panel of 59 in-house assembled patient sera pre-screened for HIV-sero-status using a commercial kit (Vidas HIV Duo Quick kit, bioMérieux SA, France), were tested using the one-step (panel **A**) and two-step (panel **B**) assays. The empty and filled circles represent sera that were negative and positive, respectively, for anti-HIV-1 antibodies, as determined by the two assays.

First, we carried out the one- and two-step assays outlined above using a panel of 59 in-house human sera that had been pre-screened with the commercially available Vidas HIV Duo Quick kit, shown in Figure 3. Based on the results with this kit, 22 sera were positive and 37 negative for anti-HIV antibodies. Remarkably, these results were mirrored exactly by both the one- and two-step r-Δgp160-based TRF assays, with no discrepancy in any of the samples whatsoever.

The results with the in-house sera panel warranted additional evaluation. For this purpose, we tested several commercially procured well-characterized sera panels described earlier [20]. To begin with, the sensitivities of the one- and two-step assays were evaluated using a HIV-1 seroconversion panel consisting of 9 samples (PRB 931). Only one out of five currently used commercial kits is capable of detecting seroconversion on day 28, represented by panel member #06. The data summarized in Table 1 show that both the in-house assays picked up panel member #06 unambiguoulsy, demonstrating that they are as good as the best performing Abbott HIV-1/2 kit in this regard. The capacity to detect early seroconversion may be attributable to the inherent design of the double antigen sandwich assay format which narrows down the seroconversion window due to its ability to pick up both IgM and IgG antibodies [20,30]. The two-step assay displayed relatively higher S/Co ratios. Next, we analyzed a worldwide panel (WWRB 302) consisting of 21 HIV-1-positive, 2 HIV-2-positive and 2 HIV-negative sera, from multiple locations in Asia, Africa, Europe, and North and South America. The data are summarized in Table 2. But for panel members #1 and #11, which were missed by the one-step assay, all other HIV-positive samples were picked out by both in-house assays. These two sera, one corresponding to HIV-1 group O (panel member #01) and the other to HIV-2 (panel member #11), were scored as weakly positive by the two-step assay. On the other hand, the HIV-2-positive panel member #25 was detectable with both the assays. Given that our antigen was not designed to pick up HIV-2, these results imply cross-reactivity of r-Δgp160 with anti-HIV-2 antibodies. Importantly, the two sera in this panel (members #10 and #30), which were HIV-negative in five different commercial assays were scored as seronegative by both our in-house assays. We then evaluated the specificity of the in-house one- and two-step assays, using a viral co-infection panel (PCA 201), consisting of 9 (of which 7 were available to us) HIV-negative and 16 HIV-positive sera samples. Many of the panel members were also seropositive for hepatitis B virus (HBV), hepatitis C virus (HCV) and/or Human T cell Leukemia virus (HTLV). Both assays recognized all HIV-1 seropositive samples correctly, irrespective of the presence of antibodies to other viruses, as shown in Table 3. It is noteworthy that of the 7 HIV-negative sera tested, 6 had evidence of other infections such as HCV, HBV or HTLV. Yet, none of these were picked up by either in-house assay, demonstrating the high degree of specificity of these assays.

**Table 1:** Evaluation of r-Δgp160-based TRF assays using HIV-1 seroconversion panel (PRB 931)

**Member ID#**	**Days since 1**^**st **^**bleed**	**Abbott**		**Gen. Sys.**		**Organon Teknika HIV**^a^	**TRF assays**^b^	
		**HIV1**^a^	**HIV1/2**^a^	**HIV1**^a^	**HIV1/2**^a^		**r-Δgp160 two-step**	**r-Δgp160 one-step**
01	0	0.2	0.1	0.2	0.1	0.3	0.9 (−)	0.5 (−)
02	2	0.2	0.1	0.1	0.1	0.3	0.8 (−)	0.5 (−)
03	7	0.2	0.1	0.2	0.1	0.4	0.8 (−)	0.4 (−)
04	9	0.2	0.1	0.2	0.1	0.3	0.8 (−)	0.5 (−)
05	15	0.2	0.1	0.2	0.1	0.3	0.7 (−)	0.5 (−)
06	28	0.9	6	0.3	0.4	0.6	17.2 (+)	5.6 (+)
07	33	3.9	>18.7	0.8	1.1	2.3	15.9 (+)	4.3 (+)
08	35	5.7	>18.7	1.3	1.9	3.1	11.9 (+)	3.3 (+)
09	42	10.5	>18.7	2.9	4	4.6	13.8 (+)	2.9 (+)

^a^Values indicate S/Co ratios, provided by the panel supplier using the indicated commercial EIA kits. S/Co values ≥ 1.0 are considered as positive.

^b^Values indicate S/Co ratios obtained using r-Δgp160-based TRF assays. The results are indicated in parentheses. Samples with S/Co values < 1.0 are designated as negative (−) and those with values ≥ 1.0 are designated as positive (+).

**Table 2:** Evaluation of r-Δgp160-based TRF assays using worldwide HIV performance panel (WWRB 302)

**Member ID#**	**Origin**	**Gtp**^a^	**Abbott**		**Gen. Sys.**		**OT HIV1**^b^	**TRF assays**^c^	
			**HIV1**^b^	**HIV1/2**^b^	**HIV1**^b^	**HIV1/2**^b^		**r-Δgp160 two-step**	**r-Δgp160 one-step**
01	Spain	O	1.1	1.8	0.8	5.6	1.3	1.3 (+)	0.6 (−)
02	Ghana	A	>11.5	>16.1	6.9	8.7	7.0	94.0 (+)	9.2 (+)
03	Ghana	G	>11.5	>16.1	7.1	8.8	7.2	164.8 (+)	41.6 (+)
04	Ghana	G	>11.5	>16.1	7.1	8.8	6.5	138.0 (+)	4.1 (+)
05	Ghana	A	>11.5	>16.1	7.1	8.7	7.0	253.6 (+)	82.2 (+)
06	Ghana	G	>11.5	>16.1	6.9	8.8	7.1	91.4 (+)	15.1 (+)
08	Ivory Coast	G	>11.5	>16.1	6.9	8.7	6.7	73.0 (+)	4.4 (+)
09	Ivory Coast	A	>11.5	>16.1	6.9	8.6	6.5	107.8 (+)	3.9 (+)
10	Ivory Coast	Neg	0.4	0.2	0.1	0.4	0.4	0.7 (−)	0.4 (−)
11	Mozambique	HIV-2	1.2	14.6	0.6	9.7	3.0	1.0 (+)	0.7 (−)
12	Mozambique	C	>11.5	>16.1	7.1	8.9	6.9	187.1 (+)	4.1 (+)
14	Uganda	D	>11.5	>16.1	4.5	8.5	6.2	53.3 (+)	13.8 (+)
15	Uganda	D	>11.5	>16.1	6.3	8.1	7.2	132.9 (+)	16.8 (+)
16	Uganda	D	>11.5	>16.1	7.0	8.8	6.9	154.5 (+)	7.3 (+)
17	Uganda	D	>11.5	>16.1	6.8	9.8	7.0	131.2 (+)	5.1 (+)
19	Zimbabwe	C	>11.5	>16.1	6.0	9.9	7.0	41.4 (+)	10.3 (+)
21	China	B	>11.5	>16.1	6.7	8.8	7.0	136.7 (+)	26.8 (+)
22	Thailand	E	>11.5	>16.1	7.3	9.8	7.0	100.8 (+)	7.0 (+)
24	Thailand	E	>11.5	>16.1	7.4	9.8	6.9	88.4 (+)	23.2 (+)
25	India	HIV-2	0.4	15.4	3.8	10	2.1	4.9 (+)	1.5 (+)
26	USA	D	>11.5	>16.1	7.4	9.8	7.1	85.8 (+)	48.5 (+)
27	USA	B/D	>11.5	>16.1	7.0	9.8	7.2	135.7 (+)	12.7 (+)
28	Argentina	F	>11.5	>16.1	7.0	8.9	6.8	72.5 (+)	3.0 (+)
29	Argentina	B	>11.5	>16.1	6.9	8.5	6.6	92.9 (+)	2.2 (+)
30	Argentina	Neg	0.3	0.2	0.2	0.2	0.4	0.9 (−)	0.4 (−)

^a^Genotype.

^b^Values indicate S/Co ratios, provided by the panel supplier using the indicated commercial EIA kits. S/Co values ≥ 1.0 are considered as positive. (Gen. Sys., Genetic Systems; OT, Organon Teknika).

^c^Values indicate S/Co ratios obtained using r-Δgp160-based TRF assays. The results are indicated in parentheses. Samples with S/Co values < 1.0 are designated as negative (−) and those with values ≥ 1.0 are designated as positive (+).

**Table 3:** Evaluation of r-Δgp160-based TRF assays using viral co-infection performance panel (PCA 201)

**Member ID#**	**Ortho EIA HCV**^a^	**Abbott EIA HBsAg**^a^	**OT Anti-HBc**^a^	**HTLV**		**HIV-1**		**TRF assays**^b^	
				**Abbott EIA**^a^	**GS Blots**^a^	**Abbott EIA**^a^	**Dupont blots**^a^	**r-Δgp160 two-step**	**r-Δgp160 one-step**
1	+	+	+	+	P	0.2	N/A	0.9 (−)	0.6 (−)
2	-	+	+	-	N/A	13.5	P	96.5 (+)	6.5 (+)
3	+	+	+	+	P	0.1	N/A	0.5 (−)	0.7 (−)
4	-	+	+	+	P	0.1	N/A	ND	ND
5	+	-	+	+	P	13.5	P	122.6 (+)	8.5 (+)
6	-	-	-	-	N/A	0.1	N/A	ND	ND
7	+	-	-	+	P	13.5	P	146.1 (+)	9.0 (+)
8	+	+	+	-	N/A	13.5	P	208.4 (+)	5.2 (+)
9	+	+	-	+	P	13.5	P	107.7 (+)	8.5 (+)
10	-	+	+	-	N/A	13.5	P	84.9 (+)	13.3 (+)
11	+	+	+	+	P	0.1	N/A	0.7 (−)	0.6 (−)
12	+	+	+	-	N/A	13.5	P	142.2 (+)	1.1 (+)
13	-	+	+	-	N/A	13.5	P	47.8 (+)	11.2 (+)
14	-	+	+	+	P	0.32	N/A	0.8 (−)	0.7 (−)
15	+	+	+	+	IND	0.2	N/A	0.8 (−)	0.7 (−)
16	+	-	+	+	P	13.5	P	182.5 (+)	4.5 (+)
17	+	+	+	-	N/A	13.5	P	215.4 (+)	9.1 (+)
18	+	+	-	+	P	13.5	P	48.3 (+)	3.6 (+)
19	-	+	+	-	N/A	13.5	P	88.2 (+)	9.7 (+)
20	-	+	+	-	N/A	1.1	P	3.3 (+)	2.5 (+)
21	-	+	+	-	N/A	13.5	P	128.6 (+)	3.0 (+)
22	+	+	+	-	N/A	13.5	P	140.6 (+)	16.9 (+)
23	+	+	+	+	P	0.2	N/A	0.8 (−)	0.8 (−)
24	-	-	-	-	N/A	0.1	N/A	0.8 (−)	0.8 (−)
25	-	+	+	-	N/A	13.5	P	127.1 (+)	6.0 (+)

^a^Assays performed by Boston Biomedica Inc (now SeraCare Life Sciences) using commercial kits. Values for Abbott anti-HIV EIA represent S/Co ratios, provided by the panel supplier. Results of other EIA tests, provided by panel supplier are indicated as either positive (‘+’, S/Co ≥ 1.0) or negative (‘-’, S/Co < 1.0) for simplicity. HTLV and HIV-1 EIA positive samples were also tested in blot formats using kits from GS (Genetic Systems) and Dupont, respectively. ‘P’and ‘IND’ indicate the positive and indeterminate results, respectively, depending upon the presence or absence of antigen bands in the blot assays. ‘N/A’ indicates not applicable. (‘OT’, Organon Teknika; ‘GS’, Genetic Systems).

^b^Values indicate S/Co ratios obtained using r-Δgp160-based TRF assays. The results are indicated in parentheses. Samples with S/Co values < 1.0 are designated as negative (−) and those with values ≥ 1.0 are designated as positive (+). ‘ND’ indicates ‘not determined’ due to lack of sample.

The data thus far reveal that both the one- and two-step r-Δgp160-based double antigen sandwich TRF assays performed reliably when tested on three different commercially procured sera panels. Recently, we had evaluated these same sera panels in a two-step TRF assay based on a mixture of recombinant HIV-1 and HIV-2 antigens [20]. As in the present study, the earlier reported assay was also in a double antigen sandwich format. At this point, we were interested to compare the sensitivity of the earlier two-step assay with the current one- and two-step assays. To this end, we analyzed an anti-HIV-1 low titer performance panel of 15 members (PRB 108), using all three assays. The earlier assay was done as before in a two-step format, but using a single antigen, r-HIV-1env (biotinylated antigen to capture and Eu^3+^-chelate labeled antigen to reveal), instead of the two antigen mixture. The results of this comparative analysis are summarized in Table 4. Panel member #2 was unequivocally seronegative by the three commercial EIAs: Abbott HIV-1/2, Genetic Systems HIV-1/2 and Organon Teknika HIV-1 tests. None of the three in-house TRF assays showed reactivity with this sample. Panel member #3, a borderline sample which could not be picked up by the r-HIV-1env-based assay, was identified unequivocally by both the one- and two-step r-Δgp160-based assays. It is noteworthy that this panel member is positive for HIV-1 RNA. Another borderline sample, represented by panel member #12, was undetectable by r-HIV-1env-based assay and one-step r-Δgp160-based assay, but identified as weakly positive by the two-step r-Δgp160-based assay. Four samples (panel member #9, 10, 13 and 14) that were HIV-1 RNA positive, but scored as negative by the Organon Teknika HIV-1 EIA kit, were identified as seropositive for HIV-1 by all three in-house TRF assays. Based on the results in Table 4, the order of performance of the three assays was: r-Δgp160-based two-step assay>r-Δgp160-based one-step assay>r-HIV-1env-based two-step assay.

**Table 4:** Evaluation of different TRF assays using anti-HIV-1 low titer performance panel (PRB 108)

**Member ID #**	**Abbott HIV 1/2**^**a**^	**Gen. Sys. HIV 1/2**^**a**^	**Org. Tek. HIV 1**^**a**^	**Bio-Rad HIV-1 Western Blot Result**^**b**^	**Roche PCR HIV-1 RNA test copies/ml**^**c**^	**TRF assays**^**d**^		
						**r-Δgp160 two-step**	**r-Δgp160 one-step**	**r-HIV-1env two-step**
1	5.5	>8.9	1.4	+	3x10^4^	1.9 (+)	1.8 (+)	2.0 (+)
2	0.2	0.3	0.3	-	BLD	0.8 (−)	0.6 (−)	0.7 (−)
3^e^	NA	NA	1.8	IND	5x10^1^*	3.4 (+)	3.0 (+)	0.8 (−)
4^e^	11.9	>8.9	6.3	+	9x10^4^	7.0 (+)	5.2 (+)	3.4 (+)
5	3.4	>8.9	3.2	+	5x10^1^*	2.2 (+)	1.2 (+)	3.6 (+)
6	NA	NA	1.2	IND	6x10^3^	1.8 (+)	1.1 (+)	5.9 (+)
7^f^	5.3	>8.9	1.3	+	3x10^3^	2.7 (+)	1.6 (+)	3.9 (+)
8^f^	8.0	>8.9	2.8	+	7x10^3^	3.8 (+)	2.8 (+)	4.8 (+)
9	8.5	>8.9	0.7	+	2x10^3^	1.9 (+)	1.1 (+)	7.2 (+)
10	8.6	>8.9	0.6	IND	3x10^5^	1.7 (+)	1.2 (+)	13.2 (+)
11	NA	NA	3.8	+	8x10^3^	3.2 (+)	2.0 (+)	5.4 (+)
12	1.7	2.0	0.3	-	5x10^5^	1.1 (+)	0.6 (−)	0.9 (−)
13	NA	NA	0.9	IND	2x10^5^	4.9 (+)	2.7 (+)	18.2 (+)
14^g^	NA	NA	0.4	-	3x10^5^	1.4 (+)	1.0 (+)	1.1 (+)
15^g^	14.2	>8.9	1.8	IND	>7x10^5^	5.9 (+)	3.0 (+)	8.0 (+)

^a^Values indicate S/Co ratios, provided by the panel supplier using the indicated commercial EIA kits. S/Co values ≥ 1.0 are considered as positive. (Gen. Sys., Genetic Systems; Org.Tek., Organon Teknika). ‘NA’ indicates data ‘not available’ from the panel supplier.

^b^Result provided by the panel supplier using the indicated commercial test. ‘+’ and ‘–’ indicate the presence and absence, respectively, of antigen bands in the Western blot assay. ‘IND’ indicates indeterminate result.

^C^Result provided by the panel supplier using the indicated commercial test. ‘BLD’ indicates that the RNA level is below the limit of detection. *Ultrasensitive method was used (50–75,000 copies/ml).

^d^Values indicate S/Co ratios obtained in this study, using the in-house TRF assays. The results are indicated in parentheses. Samples with S/Co values < 1.0 are designated as negative (−) and those with values ≥ 1.0 are designated as positive (+).

^e^PRB 108–4 is a bleed from the same donor as PRB 108–3 collected 17 months later.

^f^PRB 108–8 is a bleed from the same donor as PRB 108–7 collected 6 days later.

^g^PRB 108–15 is a bleed from the same donor as PRB 108–14 collected 7 days later.

**Figure 4:**
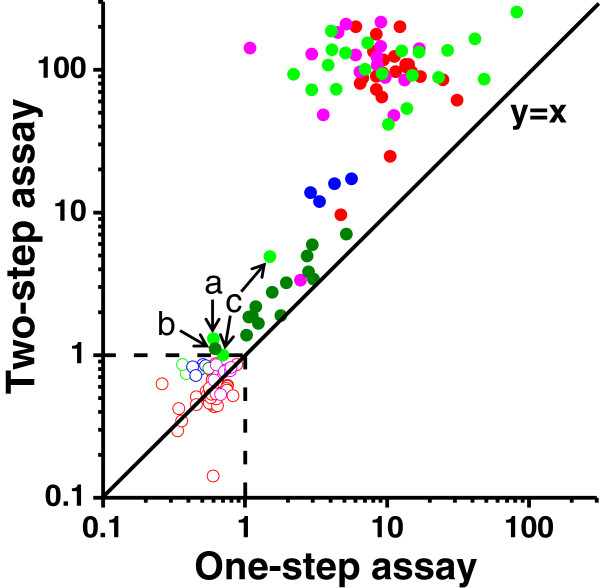
**Comparison of the one- and two-step double antigen sandwich TRF assays.** The S/Co values of all sera (n=131) analyzed in this study, using the one-step (*x* axis) and two-step (*y* axis) assay formats are included in this comparative analysis. Each circle in this plot represents one sample. The sera represented members of the in-house (red), PRB 931 (blue), WWRB 302 (green), PCA 201 (magenta) and PRB 108 (olive) panels. Filled circles represent anti-HIV positive and empty circles represent anti-HIV negative samples, as determined by the commercial tests described in the text. The dashed horizontal and vertical lines represent the cutoffs (at S/Co=1) for the two assays. Commercial panel members #1 (panel WWRB 302), #12 (panel PRB 108) and member #s 11 and 25 (panel WWRB 302), discussed in the text are indicated by ‘a’, ‘b’ and ‘c’, respectively.

Collectively the data presented so far indicate that the two-step r-Δgp160-based assay performs better as an HIV-1 diagnostic test. To delineate this aspect more clearly, we compared the S/Co values obtained for the entire collection of sera analyzed in this study (n=131), using the one- and two-step r-Δgp160-based assays. This is presented in Figure 4. The mean S/Co value for the HIV positive serum samples (n = 79) using the two-step r-Δgp160-based assay was 83.1 (95% CI: 69–97.2). The corresponding value for one-step r-Δgp160-based assay was 9.7 (95% CI: 7.1-12.3). Between the two assays, the mean S/Co value was significantly higher in the two-step assay than in one-step assay (p<0.001). We interpret this as a manifestation of the high-dose Hook effect [31], wherein sera with high titers of anti-HIV antibody, in the context of the one-step assay, sequester the tracer antigen, r-Δgp160-Eu^3+^, thereby precluding effective double antigen sandwich formation, leading to lowered S/Co values. This anomaly is eliminated in the two-step assay by introducing an intermediate washing step which removes excess antibody after the initial capture step, before addition of the tracer antigen. However, one-step assay saves time consumed in an additional incubation step.

## Conclusions

This study has shown the feasibility of overcoming the barrier to HIV-1 gp160 expression in *E. coli*. It has been demonstrated that deletion of discrete hydrophobic regions can result in the expression of near full-length gp160 in *E. coli* hosts, in reasonably high yields. Double antigen sandwich assays in conjunction with TRF-based detection unequivocally established the diagnostic potential of the *E. coli*-expressed r-Δgp160 antigen. The r-Δgp160 antigen-based one- and two-step assays, were compared with several commercial HIV detection assays using a collection of 131 sera, and found to perform as well or better. Both the assays detected early seroconversion on par with the best commercial kit and in general manifested a high degree of sensitivity and specificity. The robust performance of the one-step assay suggests its potential in envisaging a rapid point-of-care assay. The two-step assay manifested a significantly higher mean S/Co value than the one-step assay, presumably by eliminating the Hook effect. The r-Δgp160 antigen provides the basis for a highly sensitive and specific HIV-1 diagnostic assay that has the potential to cut down test cost by lowering the cost of antigen production. In conjunction with luminescent lanthanide-doped inorganic nanoparticles e.g. up-converting phosphors to label the tracer antigen which can be detected with an inexpensive reader, r-Δgp160 antigen may pave the way for an affordable test in resource-poor regions where HIV-1 is prevalent.

## Abbreviations

AIDS: Acquired immunodeficiency syndrome; aa: Amino acid; BAP: Biotin acceptor peptide; CI: Confidence interval; EIA: Enzyme immunoassay; gp160: Glycoprotein 160; r-Δgp160: Recombinant HIV-1 gp160 lacking hydrophobic regions as shown in Figure 1; r-Δgp160-Eu^3+^: r-Δgp160 labeled with Eu^3+^ chelate; HBV: Hepatitis B virus; HCV: Hepatitis C virus; HIV-1: Human immunodeficiency virus type 1; HTLV: Human T cell leukemia virus; IPTG: Isopropyl-β-D-thiogalactopyranoside; NAP: Nucleic acid purification; SA: Streptavidin; S/Co: Signal-to-cutoff ratio; SD: Standard deviation; Trx: Thioredoxin; r-Trx-BAP-Δgp160: r-Δgp160 with N-terminal Trx-BAP fusion; r-Δgp160-Bio: *in vitro* (chemically) biotinylated r-Trx-BAP-Δgp160; TRF: Time resolved fluorometry.

## Competing interests

The authors declare that they have no competing interests.

## Authors' contributions

SMT, SKN, TSa and SK conducted the experiments. SMT, SS, TSo, KP and NK designed the study and wrote the initial draft. SMT and SS wrote the final manuscript. All the authors read and approved the manuscript.

## Pre-publication history

The pre-publication history for this paper can be accessed here:

http://www.biomedcentral.com/1471-2334/12/325/prepub
